# High mobility approaching the intrinsic limit in Ta-doped SnO_2_ films epitaxially grown on TiO_2_ (001) substrates

**DOI:** 10.1038/s41598-020-63800-3

**Published:** 2020-04-22

**Authors:** Michitaka Fukumoto, Shoichiro Nakao, Kei Shigematsu, Daisuke Ogawa, Kazuo Morikawa, Yasushi Hirose, Tetsuya Hasegawa

**Affiliations:** 1 Department of Chemistry, The University of Tokyo, 7-3-1 Hongo, Bunkyo-ku, Tokyo, 113-8654 Japan; 2 Kanagawa Institute of Industrial Science and Technology (KISTEC), 705-1 Shimoimaizumi, Ebina, Kanagawa 243-0435 Japan; 3 Laboratory for Materials and Structures, Tokyo Institute of Technology, 4259 Nagatsuta, Midori-ku, Yokohama, 226-8503 Japan; 4 Tokyo Metropolitan Industrial Technology Research Institute (TIRI), 2-4-10 Aomi, Koto-ku Tokyo, 135-0064 Japan

**Keywords:** Electronic devices, Electronic materials

## Abstract

Achieving high mobility in SnO_2_, which is a typical wide gap oxide semiconductor, has been pursued extensively for device applications such as field effect transistors, gas sensors, and transparent electrodes. In this study, we investigated the transport properties of lightly Ta-doped SnO_2_ (Sn_1−*x*_Ta_*x*_O_2_, TTO) thin films epitaxially grown on TiO_2_ (001) substrates by pulsed laser deposition. The carrier density (*n*_e_) of the TTO films was systematically controlled by *x*. Optimized TTO (*x* = 3 × 10^−3^) films with *n*_e_ ~ 1 × 10^20^ cm^−3^ exhibited a very high Hall mobility (*μ*_H_) of 130 cm^2^V^−1^s^−1^ at room temperature, which is the highest among SnO_2_ films thus far reported. The *μ*_H_ value coincided well with the intrinsic limit of *μ*_H_ calculated on the assumption that only phonon and ionized impurities contribute to the carrier scattering. The suppressed grain-boundary scattering might be explained by the reduced density of the {101} crystallographic shear planes.

## Introduction

Tin dioxide (SnO_2_) has been extensively studied as a practical transparent oxide semiconductor in various applications such as field-effect transistors^1,2^, gas sensors^3–5^, and transparent electrodes^6–8^. Hall mobility (*μ*_H_) is a key parameter in determining the performance of such devices, and the *μ*_H_ values of bulk SnO_2_ single crystals are in the range of 70 to 260 cm^2^V^−1^s^−1^ at room temperature^9–11^. However, SnO_2_ thin films show a rather low *μ*_H_ of less than 100 cm^2^V^−1^s^−1^ even in well-optimized epitaxial films^12,13^, which limits the practical use of SnO_2_.

The lower* μ*_H_ in SnO_2_ epitaxial thin films is primarily attributable to the lack of lattice-matched substrates. Thus far, corundum Al_2_O_3_ and rutile TiO_2_ have been widely used as the substrates for the epitaxial growth^14,15^ of SnO_2_. Particularly, Al_2_O_3_, with a high thermal and chemical stability, is suitable for the growth of SnO_2_ thin films at high temperatures, but the SnO_2_ thin films deposited on Al_2_O_3_ suffer from lowered crystallinity owing to the difference between the crystal structures of the film and substrate. For example, very low *μ*_H_ values are frequently observed for epitaxial SnO_2_ films on Al_2_O_3_. TiO_2_ shares the same rutile structure as SnO_2_, but it has a relatively large lattice-mismatch with SnO_2_, which is 3.1% and 7.7% for the *a*-axis and *c*-axis, respectively. Indeed, it was reported that *μ*_H_ of the undoped SnO_2_ film with (001) orientation on TiO_2_ (001) was limited to a rather small value^16^, that is, ~40 cm^2^V^−1^s^−1^. To overcome the above-mentioned difficulty, very thick self-buffer layers^12,13^ have been employed to grow high-*μ*_H_ epitaxial SnO_2_ films on Al_2_O_3_.

Another important factor for achieving high *μ*_H_ is to control the carrier density (*n*_e_) because carriers play two competing roles in *μ*_H_; an increase in *n*_e_ enhances the screening of the Coulomb scattering potential and thus increases *μ*_H_, whereas an increased amount of dopants suppresses *μ*_H_ owing to impurity scattering. To date, much effort has been made to grow undoped^13–18^ or heavily doped^19–22^ SnO_2_ films on a wide variety of substrates. Heavily doped SnO_2_ films, albeit practically important, show a low *μ*_H_ that is dominated by impurity scattering. Attempts to pursue high* μ*_H_ in undoped SnO_2_ thin films have been unsuccessful owing to the significant carrier scattering by the grain boundary^18,23^ and dislocation^13,24^ induced by lattice-mismatched substrates. There is a possibility to realize a high mobility in the intermediate *n*_e_ region between undoped and heavily doped SnO_2_, but little attention has been paid to lightly doped^12,23^ SnO_2_ films.

In this study, we focus on lightly doped SnO_2_ thin films to achieve a high *μ*_H_. We investigated the electrical transport properties of lightly Ta-doped SnO_2_ (Sn_1−*x*_Ta_*x*_O_2_, TTO) films grown on TiO_2_ (001) substrates, which are isostructural to SnO_2_, with the smallest lattice mismatch. We found that the increase in *n*_e_ by Ta-doping dramatically enhanced *μ*_H_, probably owing to a screening of the carrier scattering by the grain boundaries and dislocations. The TTO films with *n*_e_ ~ 1 × 10^20^ cm^−3^ exhibited *μ*_H_ of 130 cm^2^V^−1^s^−1^, which is the highest among SnO_2_ films thus far reported. Moreover, this value is close to the intrinsic limit of *μ*_H_ calculated by assuming that only phonon and ionized impurities contribute to the carrier scattering.

## Results and Discussion

**Figure 1:**
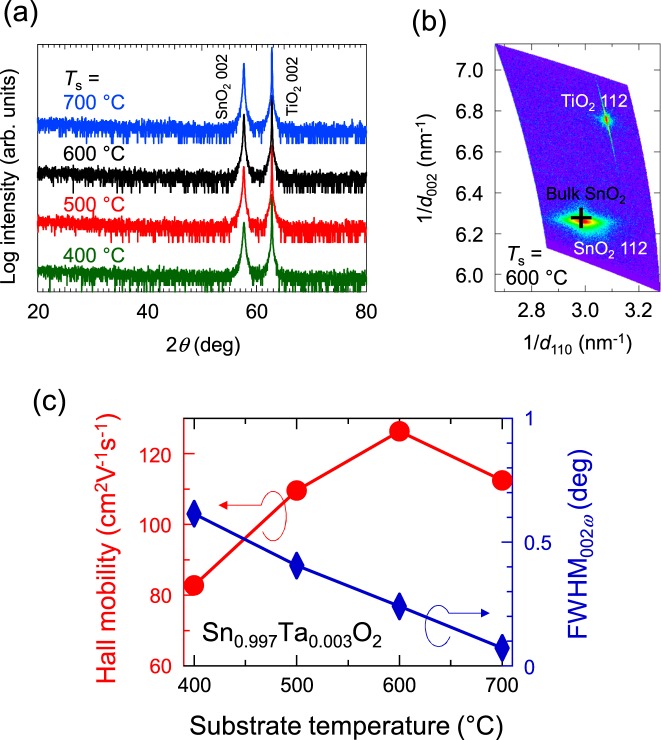
(**a**) *ω*-2*θ* X-ray diffraction patterns for Sn_1−*x*_Ta_*x*_O_2_ (TTO) films with *x* = 3 × 10^−3^ grown at various substrate temperatures (*T*_s_). (**b**) A reciprocal space map around the asymmetric 112 diffraction peaks for a TTO film grown at *T*_s_ = 600 °C. A cross represents the peak position for bulk SnO_2_. (**c**) *T*_s_ dependence of Hall mobility (*μ*_H_, circles) and full width at half maximum of rocking curve (*ω* scan) for 002 diffraction peak (FWHM_002*ω*_, diamonds) for the TTO (*x* = 3 × 10^−3^) films.

We first optimized the substrate temperature (*T*_s_) for growth of the TTO film, where the Ta content *x* was fixed at 3 × 10^−3^. Figure 1(a) shows *ω*-2*θ* X-ray diffraction (XRD) patterns for the TTO films prepared at various *T*_s_. Only 002 diffraction peaks from SnO_2_ and TiO_2_ were observed in all the films, which indicated epitaxial growth of (001)-oriented SnO_2_ films on TiO_2_ (001) without any impurity phases. Epitaxial growth of the SnO_2_ films were further confirmed by off-specular Φ-scan of 101 diffraction peaks from SnO_2_ and TiO_2_ substrates (see Supplementary Fig. S1 online). Figure 1(b) shows the reciprocal space map observed around the asymmetric 112 diffraction peak for the TTO film grown at *T*_s_ = 600 °C. The film was almost fully relaxed, as reported^16^ for undoped SnO_2_ films on TiO_2_ (001). Figure 1(c) plots the full width at half maximum of the rocking curve (*ω* scan, see Supplementary Fig. S2 online) of the 002 diffraction (FWHM_002*ω*_) as a function of *T*_s_. Notably, FWHM_002*ω*_ monotonically decreased with an increase of *T*_s_ and reached 0.07° at the highest *T*_s_ = 700 °C. This FWHM_002*ω*_ value is much smaller than that reported for the SnO_2_ film on a thick self-buffer layer^12^, that is, 0.31°, which indicated very high crystallinity of the present TTO film. A similar trend, that is, improved crystallinity at high *T*_s_, was reported in the previous research on SnO_2_ epitaxial films^23,25,26^. The TTO films grown at higher *T*_s_ tended to exhibit higher *μ*_H_, as shown in Fig. 1(c). However, a slight decrease in *μ*_H_ was observed for the film grown at *T*_s_ = 700 °C in spite of the good crystallinity. We speculate that at such high *T*_s_, interdiffusion of Sn and Ti atoms occurred at the film/substrate interface^27^, which might have caused impurity scattering and thus suppressed *μ*_H_. Hereafter we fixed *T*_s_ at 600 °C.

**Figure 2:**
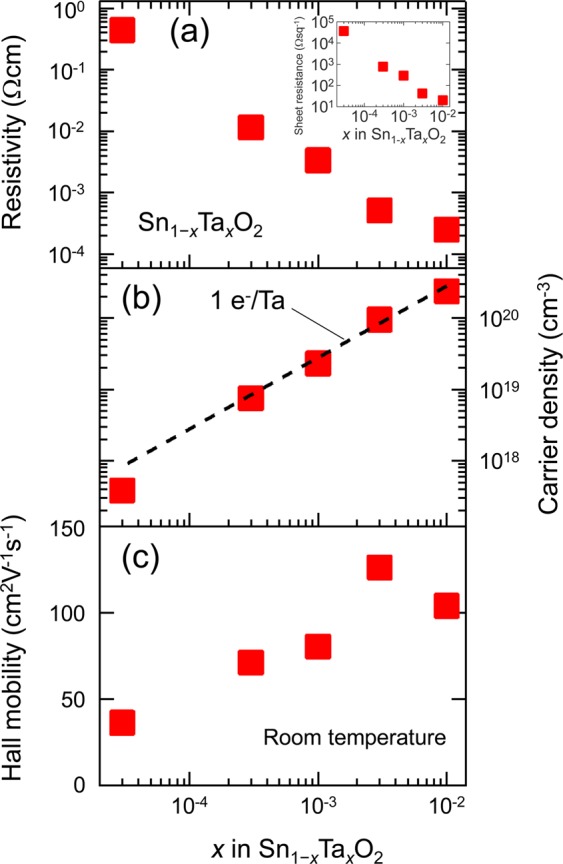
Room temperature (**a**) resistivity, (**b**) carrier density (*n*_e_), and (**c**) *μ*_H_ for the TTO films as a function of *x*. The inset of (**a**) shows sheet resistance of the films. The broken line is the expected *n*_e_ when all the doped Ta^5+^ ions substitute to the Sn^4+^ sites and generate one electron per Ta (100% doping efficiency).

Next, we investigated the dependence of the transport properties of the TTO films on *x*. As shown in Fig. 2, the TTO film with the lowest *x* = 3 × 10^−5^ showed *n*_e_ = 4 × 10^17^ cm^−3^ and *μ*_H_ = 36 cm^2^V^−1^s^−1^, which are close to those^16^ reported for undoped SnO_2_ films on TiO_2_ (001). Furthermore, *n*_e_ was proportional to *x* and lay on the line representing a 100% doping efficiency, which indicated that each Ta^5+^ ion generated one carrier electron. This implied that the lightly-doped TTO films were free from unfavourable defects such as clustered dopants^28^ and accepter-like defects^29^. Remarkably, *μ*_H_ dramatically increased with increasing *x* at *x* ≤ 3 × 10^−3^. This behavior was rationalized by assuming an enhanced screening of dislocations^13^ and/or grain boundaries^18,23^ owing to the increased *n*_e_. The TTO films with *x* = 3 × 10^−3^ (*n*_e_ ~ 1 × 10^20^ cm^−3^) exhibited the highest *μ*_H_ of 126–131 cm^2^V^−1^s^−1^, which is the highest among the *μ*_H_ values reported for undoped and doped SnO_2_ films so far. Further increase in *x* yielded a slight decrease in *μ*_H_, possibly owing to the manifestation of ionized impurity scattering, as will be discussed later. The lowest resistivity, 2.5 × 10^−4^ Ωcm, and sheet resistance, 20.2 Ωsq.^−1^, were obtained for the TTO film with *x* = 1 × 10^−2^, as shown in Fig. 2(a).

**Figure 3:**
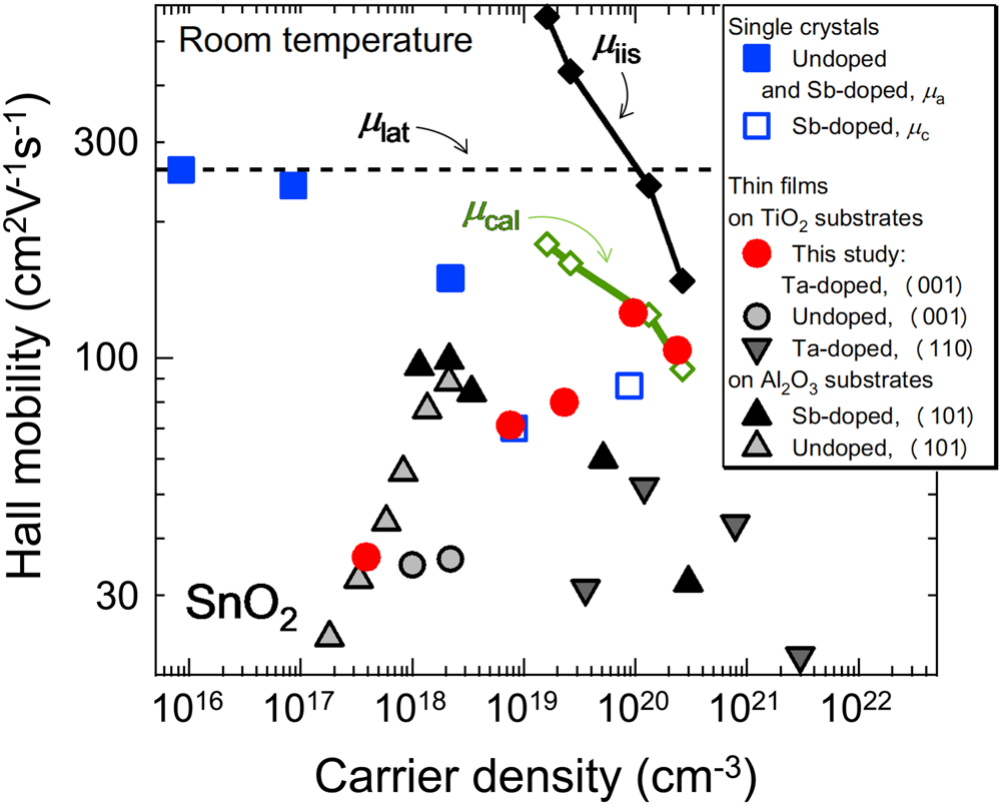
Room temperature *μ*_H_ as a function of *n*_e_ for SnO_2_ bulk single crystals (squares) and thin films [circles (present study) and triangles (literature data)]. The data for undoped single crystals in the *a*-direction (*μ*_*a*_) and Sb-doped single crystals in the *c*-direction (*μ*_*c*_) are from refs. ^9^^,11^, respectively. The data for Ta-doped (110)-, undoped (001)-, Sb-doped (101)-, and undoped (101)-films are from refs. ^23,16,12^^,13^, respectively. A solid line with diamond symbols (*μ*_cal_) represents calculated *μ*_H_ assuming that only phonon (*μ*_lat_, broken line) and ionized impurity (*μ*_iis_, solid line) scattering contribute to *μ*_H_ (*μ*_cal_^−1^ = *μ*_lat_^−1^ + *μ*_iis_^−1^).

We now discuss the transport properties of the TTO films in comparison with the literature data. Figure 3 plots *μ*_H_ against *n*_e_ for thin films^12,13,16,23^, including ours, and bulk single crystals^9,11^ of SnO_2_. The previously reported *μ*_H_ values for thin films were generally lower than those of bulk single crystals with similar *n*_e_ values. However, our TTO films with *n*_e_ ~ 1 × 10^20^ cm^−3^ exhibited a record-high *μ*_H_ (130 cm^2^V^−1^s^−1^) for thin films, which is comparable to that for a bulk single crystal with a similar *n*_e_ value. Such an extremely high *μ*_H_ value suggests that the film contained a negligibly small amount of extrinsic sources of carrier scattering, such as neutral impurities, grain boundaries, and dislocations. In other words, intrinsic sources of carrier scattering, such as phonons and ionized impurities, supposedly dominated *μ*_H_.

To test the above-mentioned hypothesis, we calculated the Hall mobility (*μ*_cal_) taking only phonon and ionized impurity scattering into account, as

$${{\mu }_{{\rm{c}}{\rm{a}}{\rm{l}}}}^{-1}={{\mu }_{{\rm{l}}{\rm{a}}{\rm{t}}}}^{-1}+{{\mu }_{{\rm{i}}{\rm{i}}{\rm{s}}}}^{-1},$$

where *μ*_lat_ is the lattice mobility associated with phonon scattering and *μ*_iis_ is the Hall mobility limited by ionized impurity scattering. For *μ*_lat_, we used a fixed value (260 cm^2^V^−1^s^−1^) observed for undoped single crystals in the *a*-direction^9^. The *μ*_iis_ value was calculated by using the Brooks–Herring–Dingle (BHD) formula^30^, which has been successfully used to analyze *μ*_iis_ for Sn-doped In_2_O_3_^31^, Al-doped ZnO^28,29^, and Nb-doped TiO_2_^32^. The BHD formula is written as

$${\mu }_{{\rm{iis}}}=\frac{24{\pi }^{3}{({{\epsilon }}_{0}{{\epsilon }}_{r})}^{2}{\hslash }^{3}{n}_{{\rm{e}}}}{{e}^{3}{m}^{\ast 2}{F}_{{\rm{ii}}}{Z}^{2}{n}_{{\rm{I}}}},$$

where *ε*_0_ is the permittivity of free space, *ε*_r_ is the relative static dielectric constant, *ħ* is the reduced Planck’s constant, *e* is the elementary charge, and m^*^ is the electron effective mass. *Z* and *n*_I_ are the charge and the density of the ionized impurity, respectively. The screening function *F*_ii_ is given by

$${F}_{ii}={\rm{l}}{\rm{n}}(1+4/x)-{(1+x/4)}^{-1}$$

with

$${\rm{\xi }}=\frac{{e}^{2}{m}^{\ast }}{\pi {{\epsilon }}_{0}{{\epsilon }}_{r}{\hslash }^{2}{(3{\pi }^{5})}^{1/3}{{n}_{{\rm{e}}}}^{1/3}}.$$

Considering the high doping efficiency, all the doped Ta was supposed to behave as singly charged ions (Ta^5+^ substituting for Sn^4+^). Although it was difficult to determine the valence state of Ta in TTO experimentally^33^ (see Supplementary Fig. S3 online), theoretical calculations^34,35^ reported that Ta exists in the pentavalent state (Ta^5+^) in TTO. Thus, we assumed *Z* = 1 and *n*_I_ = *n*_e_. Because the films in this study were (001)-oriented, we used *ε*_r*a*_ = 13.5 for *ε*_r_^36^. For m*, we used experimentally determined ${m}_{a}^{\ast }$ values as a function of *n*_e_ and their linear interpolation^37^. As shown in Fig. 3, *μ*_cal_ was higher than most of the experimental data, which indicated that the suppression of *μ*_H_ arose from carrier scattering by extrinsic sources. Notably, however, the *μ*_H_ values at *n*_e_ ≥ 9 × 10^19^ cm^−3^ (*x* = 3 × 10^−3^ and 1 × 10^−2^) in the present study agreed well with *μ*_cal_. This proved that in these high *μ*_H_ films, carrier scattering by neutral impurities, dislocations, and grain-boundaries was negligibly small compared with that by ionized impurities and phonons, and that the reduced *μ*_H_ at *n*_e_ = 2.4 × 10^20^ cm^−3^ (*x* = 1 × 10^−2^) was attributed to the increased ionized impurity scattering.

**Figure 4:**
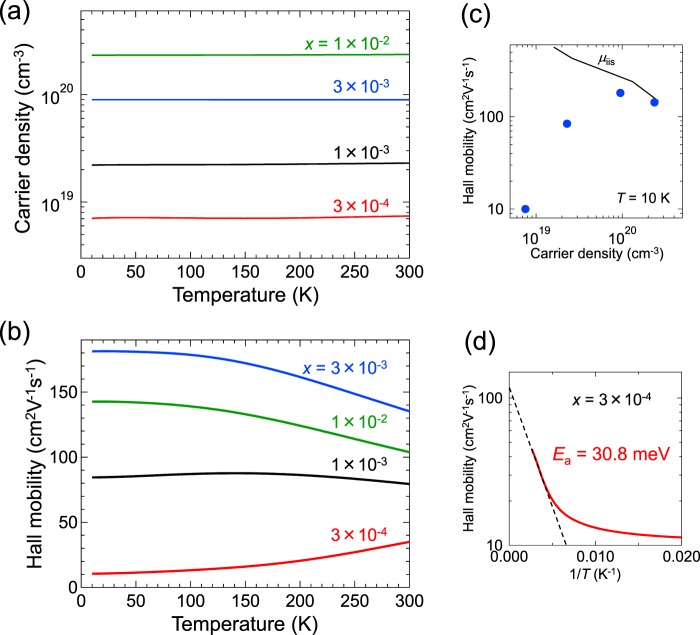
Temperature dependence of (**a**) *n*_e_ and (**b**) *μ*_H_ for the TTO films with *x* = 3 × 10^−4^ – 1 × 10^−2^. (**c**) *μ*_H_ at 10 K (circles) as a function of *n*_e_, in comparsion with *μ*_iis_ (solid line). (**d**) *μ*_H_ for the TTO film with *x* = 3 × 10^−4^ plotted against the inverse of temperature (1/*T*). The dashed line represents the least-squares fit to the Arrhenius equation, yielding an activation energy value of 30.8 meV.

To discuss the carrier scattering mechanisms in more detail, we measured temperature dependences of *n*_e_ and *μ*_H_ for in the TTO films with *x* = 3 × 10^−4^ – 1 × 10^−2^. As shown in Fig. 4(a), the *n*_e_ values were independent of temperature, indicating that the TTO films in this study were in the degenerately-doped regime. Notably, the TTO films with *x* ≥ 1 × 10^−3^ showed negative temperature coefficients of *μ*_H_ (Fig. 4(b)) around room temperature, being the specific characteristic of phonon scattering. This implies that, at room temperature, the *μ*_H_ values are dominated by phonon scattering, in consistence with the arguments based on the room temperature data (Fig. 3). At low temperature, phonon scattering is suppressed^9^, and ionized impurities are supposed to be the intrinsic sources of carrier scattering. Remarkably, as shown in Fig. 4(c), *μ*_H_ at 10 K for the TTO film with *x* = 1 × 10^−2^ (*n*_e_ = 2.4 × 10^20^ cm^−3^) agrees well with *μ*_iis_, which is known to be temperature-independent in degenerately-doped regime. This result supports the conclusion that *μ*_H_ of the film is dominated by ionized impurity scattering and phonon scattering at room temperature (Fig. 3). As *x* and thus *n*_e_ decreased, *μ*_H_ at 10 K started deviating downward from *μ*_iis_. This behaviour indicates that the TTO films with *x* < 1 × 10^−2^ contain extrinsic sources of carrier scattering, pronounced especially at low temperature. Thermal-activation-type behaviour of *μ*_H_ was observed for the TTO film with *x* = 3 × 10^−4^ (Fig. 4(d)), demonstrating that *μ*_H_ is governed by grain boundary scattering^38^ in the film, although grain-boundary scattering in SnO_2_ epitaxial films has scarcely been studied so far. Dominguez *et al*. proposed that {101} crystallographic shear planes (CSPs) in SnO_2_ films, which are induced by misfit dislocations^39^, may act like grain boundaries^18^. Similarly, we speculated that the carrier scattering at {101} CSPs was responsible for the lower *μ*_H_ than *μ*_cal_ at *n*_e_ < 9 × 10^19^ cm^−3^.

**Figure 5:**
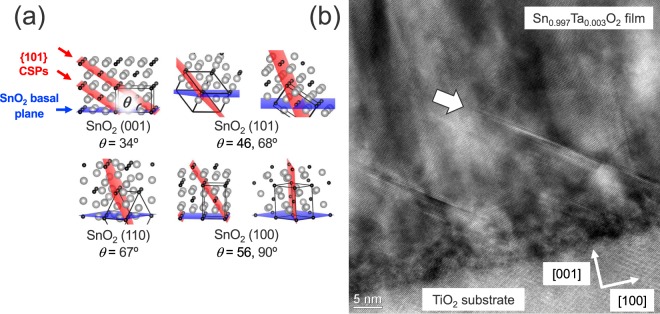
(**a**) Schematics of {101} planes, at which crystallographic shear planes (CSPs) are formed, against SnO_2_ basal planes with (001), (101), (110), and (100) orientation using the VESTA program^45^. *θ* denotes the angle between {101} and each SnO_2_ basal plane. (**b**) Cross-sectional transmission electron microscopy image of a TTO film with *x* = 3 × 10^−3^. The incident electron beam was parallel to the [010] direction. The arrow in the film indicates {101} CSP.

Judging from the complete screening by free carriers at *n*_e_ ≥ 9 × 10^19^ cm^−3^, the CSP-based grain-boundary scattering in the TTO films was supposed to be weak. We considered that lattice matching and growth orientation play an essential role in the CSP-based grain-boundary scattering as follows. Owing to the good lattice-matching to SnO_2_, the TiO_2_ (001) substrate would induce lower densities of misfit dislocations and thus CSPs in the films than other substrates^18,39^. Furthermore, the angle between {101} CSPs and the basal plane of the SnO_2_ (001) film was approximately 34°, as shown in Fig. 5(a). The shallow angle would cause termination of the {101} CSPs at the crossing point with complementary {101} CSPs^39^ at the early stage of the film growth. Indeed, as shown in Fig. 5(b), cross-sectional transmission electron microscopy (TEM) observations revealed that the TTO films on the TiO_2_ substrate had lower densities of CSPs than those on other substrates^18,39^ and that the CSPs did not reach the film surface, which supported the above-mentioned scenario. These structural characteristics can account for the lower contribution of carrier scattering at the CSP-based grain boundaries to the carrier transport in the TTO films on TiO_2_ (001). However, SnO_2_ epitaxial films on other substrates than TiO_2_ (001) have reportedly shown highly populated {101} CSPs inclined steeply to the basal planes^18,39^, as schematically illustrated in Fig. 5(a). The CSPs in SnO_2_ epitaxial films are induced by misfit dislocations, and they are not energetically favorable in bulk crystal, unlike the CSPs induced by off-stoichiometry, as seen in oxygen-deficient rutile TiO_2_ crystals^40^. Therefore, the density of CSPs decreased as the film thickness increases^18^. Nevertheless, some of the CSPs in those films survived even near the surface of the films^18^. These results suggest that the CSP-based grain-boundary scattering is more significant in the SnO_2_ epitaxial films on other substrates than TiO_2_ (001), which can account for the lower *μ*_H_ than those for the TTO films on TiO_2_ (001), as depicted in Fig. 3.

**Figure 6:**
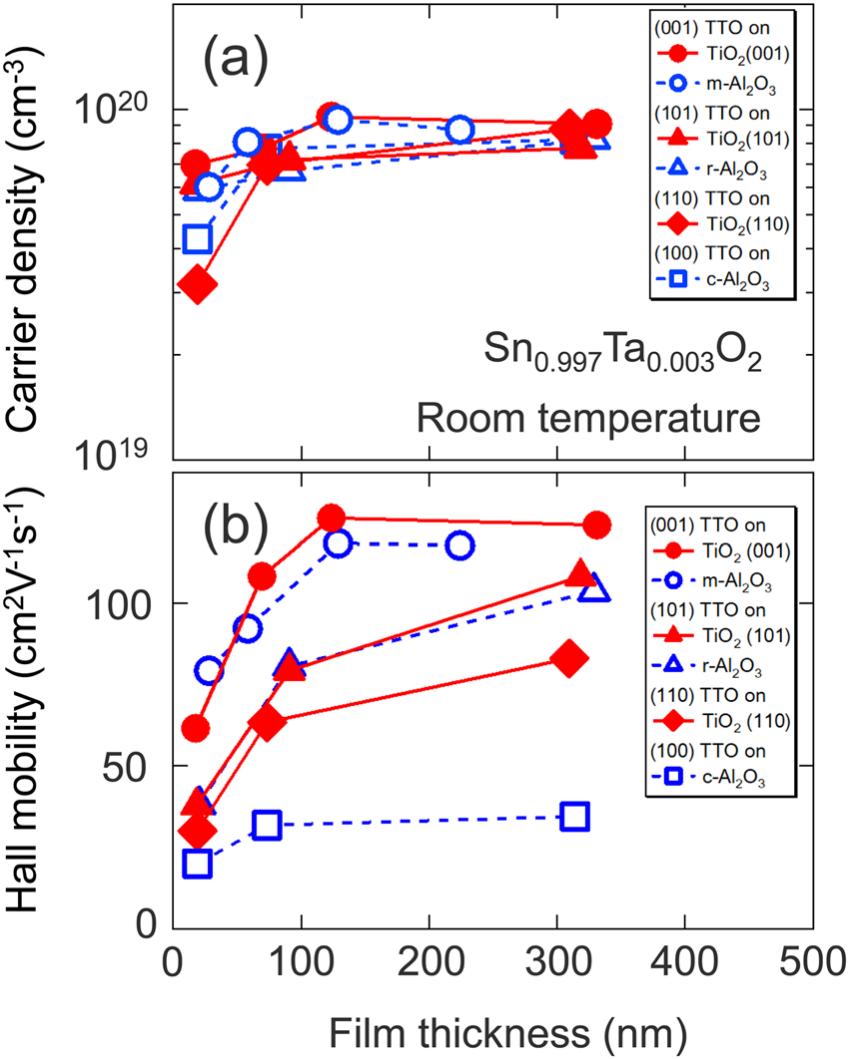
Film thickness dependence of room temperature (**a**) *n*_e_ and (**b**) *μ*_H_ for the TTO films with (001) (circles), (101) (triangles), (110) (diamonds), and (100) (squares) orientations grown on TiO_2_ (closed symbols) and Al_2_O_3_ (open symbols) substrates.

To verify the proposed model, we investigated film thickness and growth orientation dependence of *μ*_H_ for TTO films with *x* = 3 × 10^−3^ grown on various substrates^12–21,23–27,39,41,42^, (001)-, (101)-, and (110)-planes of TiO_2_, and m-, r-, and c-planes of Al_2_O_3_ substrates (see Supplementary Fig. S4 online). Figure 6 plots room temperature *n*_e_ and *μ*_H_ for the TTO films with various film orientations as a function of the film thickness. With increasing film thickness, the *μ*_H_ values increased probably owing to the synergistic effect of enlarged crystalline grains^43,44^ and reduced density of threading dislocations^24^ and {101} CSPs^18,39^. The highest *μ*_H_ was achieved for the (001)-oriented TTO films, followed in order by the (101)-, the (110)-, and the (100)-oriented ones. This behaviour can be explained by the CSP-based grain-boundary scattering because the angle between the CSP and the basal planes of the films becomes small in the same order (Fig. 5(a)). Notably, the TTO films with the same orientation showed similar *μ*_H_ values even though different kinds of substrates were used. The orientation dependence of *μ*_H_ cannot be explained by the anisotropy in electron effective mass of SnO_2_ (see Supplementary Fig. S5 online). It was suggested that {101} CSPs play a significant role in the carrier transport in the TTO epitaxial thin films.

## Summary

We investigated the transport properties of Sn_1−*x*_Ta_*x*_O_2_ (TTO) films with *x* = 3 × 10^−5^–1 × 10^−2^ epitaxially grown on TiO_2_ (001) substrates. The *n*_e_ values for the TTO films were almost equal to the concentrations of Ta dopants, which demonstrated the very high doping efficiency of Ta. The *μ*_H_ values of the TTO films with *n*_e_ ≥ 9 × 10^19^ cm^−3^ (*x* ≥ 3 × 10^−3^) agreed well with the intrinsic limit of *μ*_H_ assuming that only phonon and ionized impurities contributed to carrier scatterings. Negligible contribution of the grain-boundary scattering to *μ*_H_ might arise from a reduced density of CSPs. The TTO films with *n*_e_ ~ 1 × 10^20^ cm^−3^ (*x* = 3 × 10^−3^) exhibited a very high *μ*_H_ of 130 cm^2^V^−1^s^−1^, which is the highest among SnO_2_ films thus far reported. The *μ*_H_ values for the TTO (*x* < 3 × 10^−3^) films rapidly decreased with a decrease of *x*, which suggested a weakened screening of dislocation and/or grain-boundary scatterings owing to the decreased *n*_e_.

## Methods

TTO films with a thickness of 100–120 nm, with *x* = 3 × 10^−5^–1 × 10^−2^, were grown on TiO_2_ (001) substrates by pulsed laser deposition (PLD) with a KrF excimer laser. TTO films with *x* = 3 × 10^−3^ were grown (001)-, (101)-, and (110)-planes of TiO_2_, and m-, r-, and c-planes of Al_2_O_3_ substrates. The repetition rate and the fluence of the laser were set at 2 Hz and 1–2 J ∙ cm^−2^, respectively. The typical growth rate was 0.14–0.17 Å per shot. Sintered pellets of TTO with *x* = 3 × 10^−4^–1 × 10^−2^ were used as PLD targets. TTO films with *x* = 3 × 10^−5^ were fabricated by alternating ablation^23^ of a commercial undoped SnO_2_ (4 N purity, Toshima MFG) target and a TTO pellet with *x* = 3 × 10^−4^. In this study, nominal *x* values were used to represent the chemical compositions of the films because stoichiometric transfer of Ta from the targets to the films has been reported for TTO films grown under a similar condition^23^. The base pressure of the PLD chamber was maintained at 3 × 10^−9^ Torr. Oxygen partial pressure and *T*_s_ during film growth were 1 × 10^−2^ Torr and 400–700 °C, respectively. Crystal structure and crystallinity were evaluated by XRD measurements using a four-circle diffractometer (Bruker AXS, D8 DISCOVER). The cross-sectional microstructure of the films was observed by using a transmission electron micrscope (FEI, Titan Cubed G2 60-300) operated at 300 kV. Hall effect and resistivity were measured by using a standard six-terminal method. The Hall-bar width and the distance between voltage terminals for four-probe measurements were 1 mm and 2.4 mm, respectively. Ag or In electrodes were used for ohmic contacts. A laboratory constructed system equipped with a 2 T electromagnet was used for room temperature measurements. Current–voltage characteristics and Hall voltage-magnetic field characteristics were measured repeatedly (at least twice) to confirm the reliability and reproducibility of the measurements. Temperature dependence of the transport properties was measured with a commercially available system (Quantum design, physical properties measurement system (PPMS Model 6000)).

## Supplementary information

Supplementary information.

## Data Availability

The datasets during the current study are available from the corresponding author on reasonable request.
