# Supplementary material for: High mobility approaching the intrinsic limit in Ta-doped SnO2 films epitaxially grown on TiO2 (001) substrates
Source: Sci Rep. 2020 Apr 22;10:6844. doi: 10.1038/s41598-020-63800-3 (PMC7176643; doi:10.1038/s41598-020-63800-3)
Supplement: Supplementary file 1 — Supplementary information. [file 41598_2020_63800_MOESM1_ESM.doc]

Supplementary Information

High mobility approaching the intrinsic limit in Ta-doped SnO_2_ films epitaxially grown on TiO_2_ (001) substrates

Michitaka Fukumoto,^1^ Shoichiro Nakao,^1,2 *)^ Kei Shigematsu,^2, 3^ Daisuke Ogawa,^4^ Kazuo Morikawa,^4^ Yasushi Hirose,^1,2^ and Tetsuya Hasegawa,^1,2^

1. Department of Chemistry, The University of Tokyo, 7-3-1 Hongo, Bunkyo-ku, Tokyo 113-8654, Japan

2. Kanagawa Institute of Industrial Science and Technology (KISTEC), 705-1 Shimoimaizumi, Ebina, Kanagawa 243-0435, Japan

3. Laboratory for Materials and Structures, Tokyo Institute of Technology, Yokohama 226-8503, Japan

4. Tokyo Metropolitan Industrial Technology Research Institute (TIRI), 2-4-10 Aomi, Koto-ku, Tokyo 135-0064, Japan

^*)^ Correspondence and requests for materials should be addressed to S.N. (email: nakao@chem.s.u-tokyo.ac.jp)

Fig. S1 Off-specular Φ-scan of 101 diffraction peak from Sn_1−_*_x_*Ta*_x_*O_2_ (TTO) film with *x* = 3 × 10^−3^ and 101 diffraction from TiO_2_ substrate. The epitaxial relationship is SnO_2_(001)[100] //TiO_2_(001)[100].

Fig. S2 Rocking curves (*ω* scan) of the 002 diffraction for TTO films with *x* = 3 × 10^−3^ grown at various substrate temperatures (*T*_s_).

Fig. S3 Ta4d 5/2 photoemission spectrum of TTO film with *x* = 0.01 measured by an X-ray photoemission spectrometer (JEOL, JPS-9010MC). The Ta4d 5/2 emission line was too weak to determine the valence state of Ta experimentally.

Fig. S4 *ω*-2*θ* X-ray diffraction patterns for 300-nm-thick TTO films with *x* = 3 × 10^−3^ grown on various substrates. The asterisk symbols denote substrate peaks.

Fig. S5. (a) Hall mobility, (b) angle between current direction (*I*) and [001], (c) angle between basal plane and {101} planes, and (d) carrier density of TTO epitaxial films with various growth orientation. Inset of (a) illustrates the relation between *I* and in-plane orientation of three domains in the TTO film grown on c-Al_2_O_3_.
